# Understanding and Exploiting Biological Mechanisms of Radiosensitization Using High Atomic Mass Nanomaterials

**DOI:** 10.3390/nano16080457

**Published:** 2026-04-13

**Authors:** Beatriz Mateo, Khushbu Patel, Sean V. Murphy, Ravi Singh

**Affiliations:** 1 Department of Cancer Biology, Wake Forest University School of Medicine, Winston-Salem, NC 27157, USA; bea.mateo@wfusm.edu (B.M.); khushbu.patel@wfusm.edu (K.P.); 2 Department of Regenerative Medicine, Wake Forest University School of Medicine, Winston-Salem, NC 27157, USA; sean.murphy@advocatehealth.org; 3 Atrium Health Wake Forest Baptist Comprehensive Cancer Center, Winston-Salem, NC 27157, USA

**Keywords:** radiation therapy, cancer, nanoparticle, X-ray, cell stress, proteotoxicity, autophagy, ferroptosis

## Abstract

Radiation therapy is an essential mode of treatment for cancer, but it is limited by resistance, potential damage to healthy tissue, and inefficacy in later-stage cancers. To overcome these limitations, nanoparticles made from high atomic number (Z) atoms, such as silver (AgNPs), gold (AuNPs), and hafnium oxide (HfONPs), have been investigated for their ability to increase radiation dose deposition in cancer cells. Historically, it is believed that radiation dose enhancement primarily is achieved by physical mechanisms like photoelectric and Compton effects. Based upon these mechanisms, the usage of high Z nanoparticles would be expected to have relatively small dose enhancements and a lack of selectivity towards cancer cells under most clinical irradiation conditions. However, high Z nanoparticles exhibit very promising radiosensitizing effects that cannot fully be accounted for by physical effects, suggesting underlying biological interactions with relevant cellular processes caused by the nanoparticles themselves. Specifically, high Z nanoparticles can directly damage proteins and vesicles involved in degradation pathways (e.g., lysosomes and autophagosomes) and induce lipid peroxidation. The observed radiosensitizing effects of high Z nanoparticles may be caused by the sublethal cytotoxic responses of cancer cells to the nanomaterials themselves and are significantly greater than expected, based upon the macroscale physical dose increases in radiation deposition due to the presence of nanomaterials. This review critically analyzes the underlying biological mechanisms that could contribute to the enhancement of radiation effects by these nanomaterials.

## 1. Introduction

Targeted ionizing radiation exposure can destroy cancer cells and is a fundamental therapeutic tool in oncology [1]. Cancer cells are sensitive to radiation due to their high proliferative rates, elevated generation of reactive oxygen species, and dysfunctional DNA damage repair mechanisms [2]. However, the use of radiotherapy for cancer treatment poses toxicity concerns due to unintended damage to adjacent or intervening healthy tissues [3]. Therefore, it is important to find a balance between exposure and frequency of radiation to minimize detrimental effects on surrounding normal cells. Advanced radiation delivery techniques including Volumetric Arc Therapy (VMAT) and Intensity Modulated Radiotherapy (IMRT) have improved conformal radiation delivery, but limits to dose escalation are still needed to minimize radiotherapy-related risks to nearby or intervening organs. This persistent issue catalyzed interest in the exploration of radiosensitizers with the objective of enhancing patient outcomes by identifying sensitizers that increase the susceptibility of cancer cells to radiation [4,5,6].

Nanoparticles are materials that are smaller than 100 nm in at least one dimension, and they have unique properties that distinguish them from bulk materials, such as distinct chemical reactivity, biological mobility, and energy absorption. The use of nanotechnology is enabling promising advances in cancer imaging, diagnosis, and treatment [7]. One area of research is the use of nanoparticles to enhance the effectiveness of radiation therapy. It was hypothesized that nanoparticles made from high atomic number (high Z) elements, when used in radiotherapy, could enhance radiation dose deposition in tumors due to their large interaction cross-section with ionizing radiation [8,9,10,11,12,13]. The delivery of high Z nanoparticles to tumors has the potential to selectively increase the dose of radiation absorbed by cancer cells that take up the nanoparticles without the need to escalate the total tissue exposure to radiation. Despite successes in both preclinical and clinical settings, the potential of high Z nanomaterials as radiation sensitizers has not yet been fully realized. Major limitations include: (1) challenges in the systemic delivery of nanoparticles for selective tumor uptake [14]; (2) experimental evidence indicating that the radiosensitizing effects of various nanomaterials are heterogeneous; and (3) a lack of correlation between dosimetry calculations and observed biological assessments of dose enhancement [15]. As we will highlight in this review, the observed radiosensitizing effects of high Z nanoparticles may be caused by sublethal cytotoxic responses of cancer cells to the nanomaterials themselves and can be significantly greater than would be expected based upon the macroscale physical dose increases in radiation deposition due to the presence of nanomaterials. Several hypotheses have been proposed to explain the experimentally observed biological effects of nanoparticle radiosensitization. The first posits that at the micro- and nanoscale, the radiation dose distribution near high Z nanoparticles is heterogeneous, and can be extremely large, resulting in substantially more damage to nearby organelles and biomolecules [16]. A second hypothesis centers around the enhanced production of reactive oxygen species (ROS) caused by the internalization of high Z nanoparticles [17]. A third hypothesis, and the focus of this review, is that the presence of high Z nanomaterials in cells impacts cell type-dependent cytotoxic or stress responses, which can potentially overwhelm cellular defense mechanisms and enhance radiosensitivity. Differences in baseline levels of sensitivity to specific stresses or the magnitude of the stress response could explain why some cancers appear to be more amenable to radiosensitization by high Z nanoparticles compared to others.

This review critically analyzes the potential underlying biological mechanisms that could contribute to the enhancement of radiation effects by these nanomaterials and is not intended to be a comprehensive evaluation of all nanomaterials currently being studied as radiosensitizers. Here, we examine three major types of high Z nanomaterials with solid cores made from gold (AuNPs), silver (AgNPs), and hafnium oxide (HfONPs) that are used for radiation sensitization. We note that there are many more high Z nanomaterials being explored for this purpose including ones containing gadolinium [18] or bismuth [19], but these represent a more heterogeneous set of chemical compositions and structures and therefore are beyond the scope of this review. We describe physical mechanisms of action and contrast these against potential biological mechanisms of action that can enhance cancer selectivity and therapeutic response. Understanding and exploiting cancer-specific vulnerabilities to high Z nanomaterials may enable radiosensitization without equivalently sensitizing normal tissue to radiation.

## 2. Overview of Nanoparticle Use in Cancer Therapy and Diagnosis

A significant focus of research in cancer nanotechnology involves the use of nanoparticles as drug delivery vehicles for cancer treatment. This approach facilitates the modification of the pharmacokinetic characteristics of encapsulated drugs, thereby enhancing therapeutic efficacy while concurrently minimizing adverse effects [20]. The tunable size, surface chemistry, and ability to encapsulate or conjugate therapeutic agents allow for precise spatiotemporal control over drug release by nanoparticles. This is particularly advantageous in oncology, where systemic toxicity often limits the dosing and efficacy of chemotherapeutics [21]. Nanoparticles are believed to passively accumulate in tumors via the enhanced permeability and retention (EPR) effect [22], and when functionalized with targeting ligands, they can actively engage tumor-specific receptors to improve intracellular delivery and minimize off-target effects [23]. However, there is extensive debate regarding the relative importance of EPR [24]. What is clear is that nanoparticles can alter the bioavailability, biodistribution, and toxicity of free drugs in ways that can improve patient outcomes. Several nanoparticle-based chemotherapeutics, such as liposomal doxorubicin (Doxil^®^) [25] and albumin-bound paclitaxel (Abraxane^®^) [26], have received FDA approval for the treatment of multiple cancers, validating the clinical feasibility of nanoparticle drug delivery systems. Nanoparticles can improve tumor-targeted delivery and potentially enhance the anti-tumor immune responses caused by encapsulated drugs compared to free drugs [27]. The modularity of nanoparticle design also opens the door for combination therapies such as radiosensitizers co-loaded with chemotherapeutics or immunomodulators, enabling synergistic effects and multimodal treatment strategies.

Nanoparticle-based drug carriers have made the most impact to date, but high Z nanoparticles also demonstrate clinical utility, most notably in diagnostic imaging. Metal nanomaterials are employed as contrast agents in X-ray and computed tomography (CT) imaging due to their superior X-ray attenuation compared to conventional iodine-based agents [28,29]. AuNPs, such as AuroVist™, were investigated for preclinical imaging applications, offering enhanced contrast, prolonged circulation, and tumor-specific accumulation [29]. There are limited clinical studies involving AgNPs for cancer treatment, but their potential was demonstrated in a case study in which a stage four metastatic head and neck squamous cell carcinoma patient who did not respond to conventional treatments began a self-administered AgNP solution treatment by ingestion for three months. The patient achieved a complete remission of the cancer and experienced no adverse reactions to AgNPs [30]. It is important to note that this single report on cancer therapy using AgNPs is largely anecdotal, and no broad conclusions can be drawn from it. What is clear is that when properly designed and dosed, nanoparticles can broaden the therapeutic window of radiotherapy, leading to greater efficacy [31]. Clinical studies of HfONPs indicate improved tumor response rates for patients treated with HfONPs and radiation in various cancers including soft-tissue sarcoma [32] and head and neck cancer [33], and active clinical trials are ongoing for the treatment of non-small-cell lung cancer (NSCLC; ClinicalTrials.gov ID NCT04505267) as well as to assess the effectiveness of HfONPs combined with radiation and immune checkpoint inhibitors (ClinicalTrials.gov ID NCT03589339). Additionally, gadolinium (Gd)-containing nanoparticles, most notably AGuIX [34], have been assessed as radiosensitizers and have advanced to clinical trials. Unlike AuNPs, AgNPs, and HfONPs, these Gd-containing nanoparticles are assemblies of Gd chelates and contain only a few Gd atoms. This type of nanomaterial is excluded from further analysis because the focus of this review is on nanomaterials with a solid core of high Z atoms with minimal complexity. The advantages of using nanomaterials designed with a solid core and minimal surface passivation are the simplicity of the particle design and ease of scale-up.

There remains an enormous opportunity to expand the use of nanoparticles for radiation therapy. However, two main assumptions have limited the design of current cancer nanomedicines. The first is that the nanoparticle itself must be biologically inert (non-toxic). The second is that nanoparticle toxicity mechanisms identified in one cell can be broadly extrapolated to other cell types. Following this dogma has led to the stagnation of nanomaterial translation [24]. Cancer therapy is inherently toxic, and cancer is a highly heterogeneous collection of diseases, each with distinct vulnerabilities. To be useful, there must be a window between the toxicity of the treatment to cancer cells/tumors and the toxicity to normal cells/tissue. Identification of which cancers are most likely to respond to the specific type of toxicity induced by a particular nanomaterial used as a radiosensitizer provides an opportunity to increase the efficacy/toxicity ratio. Current research, discussed below, is beginning to unravel this complex issue.

## 3. Physical Mechanisms of High Z Nanomaterial Radiosensitization

High Z nanoparticles are believed to act as radiosensitizers by increasing the radiation dose deposited locally to cells. The dose enhancement factor (DEF) is the ratio of the dose delivered to cells with and without the nanoparticles. The DEF depends on the material composition and concentration and the energy of the irradiating X-ray beam. The dose enhancement ratio (DER) is a measure of the relative biological effectiveness of treatment in the presence of a radiosensitizer compared to treatment in the absence of a radiosensitizer. It is defined as the dose of radiation alone needed to achieve a specific biological effect divided by the dose of radiation in the presence of a radiosensitizer needed to achieve an equivalent effect. The two major types of physical interactions of X-rays with high Z nanomaterials are the photoelectric effect or the Compton effect (scattering). Here, we examine the opportunities and limitations of high Z nanoparticles in the context of these physical mechanisms of radiosensitization.

### 3.1. Photoelectric Effect

**Figure 1:**
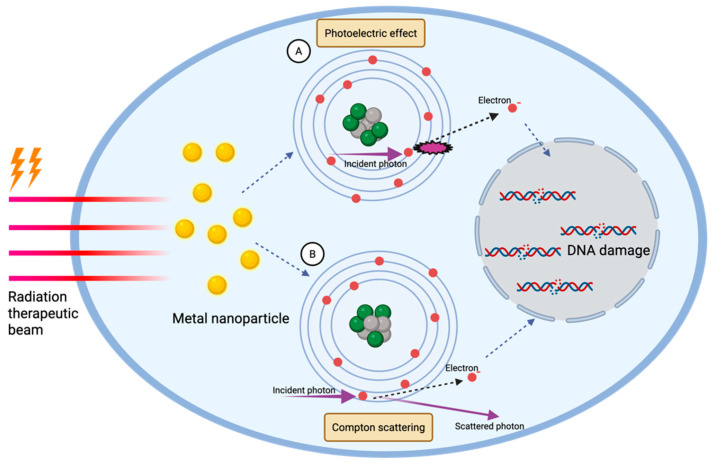
**Physical mechanisms of high Z nanomaterial radiosensitization**. (**A**) In the photoelectric effect, an incident photon interacts with the metal nanoparticle and is completely absorbed, resulting in the ejection of a high-energy electron from the atom. These emitted electrons can subsequently induce DNA damage and damage to other biomolecules (proteins and lipids) within the cell. (**B**) In Compton scattering, the incoming photon transfers part of its energy to an electron, ejecting it from the atom, while the photon itself is deflected with reduced energy. Both processes increase local electron production, amplifying DNA, protein, and lipid damage, and thereby enhancing the radiosensitization effect of high Z nanoparticles.

The fundamental concept that has guided research regarding the enhancement of localized radiation doses by high Z nanoparticles is the photoelectric effect. The energy range in which the photoelectric effect predominates in tissue is 10–25 keV [35]. The photoelectric effect occurs when incoming photons (e.g., X-rays) interact with the inner shell electrons of the target atom. When a photon collides with a tightly bound electron, it can eject that electron from its orbit [36]. This ejection creates a vacancy that may be filled by an electron from a higher energy level [37]. The release of energy during this process leads to the emission of Auger electrons, which are known for their high linear energy transfer and limited range (Figure 1A). These properties enable the targeted induction of damage within tumor cells that have taken up high Z nanoparticles, which presents a means to reduce the required doses of radiotherapy and mitigate normal tissue toxicity [37]. This interaction depends on the energy of the incoming photon, as well as the atomic number of the target material; the probability of a photoelectric event increases inversely with photon energy and increases with atomic number [35]. Research comparing the mass energy absorption coefficients for high Z nanoparticles like AuNPs to soft tissue indicates that there exists a specific energy range (approximately 10–100 keV) where the difference in photon absorption between gold nanoparticles and conventional tissue becomes significantly relevant [38]. Due to low tissue penetrance, keV X-rays are suitable only for superficial tumors, and X-rays in this energy range typically are used for diagnostic rather than therapeutic purposes.

### 3.2. Compton Scattering

Most clinical irradiators generate high energy X-rays (MeV (mega-electronvolt) range). At MeV energies, X-rays have far greater tissue penetrance than those with keV energies [39]. The energy range in which Compton scattering predominates in tissue is about 25 keV–25 MeV, and therefore Compton scattering is the most common interaction occurring clinically [40]. A photon from an X-ray source collides with an outer orbital electron and ejects it. The incoming photon is also scattered, and both the scattered photon and ejected electron continue to undergo additional low energy/high probability interactions with cellular structures (Figure 1B), leading to damage and cell death [41]. The probability of Compton scattering depends on the energy of the incident photons and the electron density of the material but is independent of the Z value of the target atom [42]. To increase the probability of Compton scattering, it is necessary to use a high mass fraction of nanomaterial to increase local electron density [43,44]. At MeV energies, significant radiosensitization due to Compton scattering would not be expected at typical concentrations of high Z nanoparticles delivered to tissue for the purpose of radiosensitization [45]. For example, it is estimated that for a mass fraction of 0.1% AuNPs (1 mg of nanoparticles per gram of water) the radiation DEF would be minimal (1.002 to 1.003) [46]. Doses far greater than 10 mg/mL of AuNPs would be needed for substantial radiosensitization due to increased Compton scattering, and the high local mass concentrations needed to achieve even modest dose enhancement means that the route of administration would be limited to direct injection into the tumor. These same issues apply to other high Z nanoparticles when assessing the role of Compton scattering for increased radiation dose deposition.

### 3.3. Limitations of Physical Mechanisms of High Z Nanomaterial Radiosensitization

Multiple studies demonstrate that high Z nanoparticles are effective for enhancing therapeutic outcomes for irradiated cancer cells and tumors across the high keV and low MeV X-ray range. However, X-rays in the high keV and low MeV range primarily interact with atoms via Compton scattering, and as discussed above, the mass attenuation coefficient for Compton scattering is independent of Z and is proportional to the number of electrons per gram, which varies only 20% between low and high Z elements. This means that high Z nanoparticles delivered to tumors minimally affect the absorbed dose of ionizing radiation under most current clinical conditions [47]. Despite models based upon the mass attenuation effects of nanomaterials on X-ray dose deposition predicting minimal DEFs (see Section 3.2), the experimentally observed DERs and therapeutic outcomes are substantial (Table 1). If high-Z-nanoparticle-mediated radiosensitization exclusively relied upon physical mechanisms, there are several issues that would limit its application. First: The major premise by which high Z nanoparticles like AuNPs and HfONPs are believed to enhance local radiation dose is through the photoelectric effect, a process that occurs predominantly at low X-ray energy (in the keV (kiloelectronvolt) range) [35]. To achieve even a modest DEF, it is necessary to deliver high doses of these nanomaterials directly to tissue [48]. Second: This mechanism is not selective for cancer cells, meaning that any cell that takes up high Z nanomaterials will be subjected to an increased absorbed dose of radiation following X-ray exposure [32]. Third: As mentioned above, due to low tissue penetrance, keV X-rays are suitable only for superficial tumors, and therefore most clinical irradiators generate higher energy X-rays (MeV range) with far greater tissue penetrance [47]. Despite these limitations, experimental and clinical results suggest that radiosensitization by high Z nanoparticles is effective, and as discussed below, potentially dependent upon the nanomaterial toxicity profile and the cell type [49,50,51]. This implies that nanomaterials possess other mechanisms of radiosensitization that are independent of the physical mechanisms of radiation dose enhancement.

## 4. Efficacy of High Z Nanoparticles as Radiosensitizers

The major organic elements have relatively low atomic numbers, such as hydrogen (1); carbon (6); nitrogen (7); oxygen (8); phosphorus (15); and sulfur (16), as do key biologically prevalent metals: magnesium (12); iron (26); copper (29); and selenium (34). Nanoparticles made from elements with atomic numbers greater than those found in the human body, including silver (47), hafnium (72), and gold (79), have larger radiation cross-sections than elements naturally found in the body, which makes them suitable for enhancing radiation dose deposition. Below, we briefly examine the efficacy of these nanomaterials for enhancing the outcomes of radiotherapy. We specifically note the radiation sources used in each study and we highlight that enhanced therapeutic outcomes are observed when high Z nanoparticles are combined with either low (keV) or high (MeV) X-rays. We emphasize that the lack of dependence of high-Z-nanoparticle-based radiosensitization on X-ray energy calls into question the role of increased dose deposition on the observed biological outcomes.

### 4.1. Gold Nanoparticles

The use of AuNPs as radiosensitizers was pioneered in the early 2000s by Hainfeld and colleagues [52]. In one of their first studies, BALB/c mice were subcutaneously injected with EMT-6 murine mammary carcinoma cells, and 1.9 nm diameter spherical AuNPs were administered via tail vein injection at a concentration of 1.35 or 2.7 g Au/kg body weight. Size distribution was confirmed using electron microscopy, with dimensions specifically tailored to avoid the high liver clearance typical of larger particles while exploiting the ‘leaky’ nature of tumor vessels for better accumulation. The mice were exposed to 250 kVp X-rays at a total dose of 30 Gy. The tumors were irradiated approximately 2 min after the AuNP injection. At this X-ray energy, both photoelectric effects and Compton scattering are predicted to play a role in interactions with tissue and nanomaterials. The results showed a significant difference in tumor growth rate between the mice receiving AuNP pre-treatment and those receiving only radiation. A month after tumor challenge, all tumors in mice treated with radiation only had regrown to five times their initial size, whereas only one out of ten tumors in mice treated with the combination of AuNPs and radiation grew back. Mice treated with AuNPs alone showed no therapeutic benefit. The one-year survival rate was reported to be 86% for mice treated with AuNPs and radiation as opposed to 20% and 0% for radiation or AuNPs alone respectively. Mammography demonstrated rapid AuNP accumulation in tumors followed by a slow clearance rate. The clearance rate of the AuNPs was almost twice as fast from normal tissue than tumor tissue. Toxicity studies showed that these AuNPs were not toxic to mice, and no major side effects were reported. These data provided strong support for the use of AuNPs as radiosensitizers, and numerous studies followed [38]. Most other studies did not achieve the same degree of tumor control that was reported by Hainfeld. One possibility is that because of the short 2 min interval between the delivery of AuNPs and radiation, the AuNPs in Hainfeld’s studies remained at high concentration in the tumor blood pool resulting in enhanced delivery of radiation to the tumor blood vessels. Modeling of this mechanism of action indicates that damage to tumor vasculature likely plays a role in AuNP-mediated radiosensitization [57]. Further research will be needed to determine optimal dosing and delivery intervals between AuNPs and radiation to maximize therapeutic efficacy.

Most clinical irradiators use beams with MeV energies where Compton scattering predominates. However, some studies show that AuNPs act as radiosensitizers for X-rays at both keV and MeV energies, indicating that the physical mechanisms of dose enhancement do not fully account for radiosensitizing effects. For example, using similarly sized AuNPs to those used in the study described above (1.9 nm, spherical AuNPs commercially sold as Auravist), Jain et al. expanded on Hainfeld’s findings by demonstrating dose enhancement effects for both 160 keV and 6 MeV X-ray beams for AuNP-treated breast cancer cells (MDA-MB-231) [49]. The physical mechanisms of dose enhancement frequently cause increased DNA damage. However, no significant difference in 53BP1 foci, a marker of DNA double strand breaks, were found in AuNP-treated cells before or after irradiation in comparison to cells treated with radiation alone. In the same study, AuNPs also potentiated the effects of the radiomimetic agent bleomycin in the absence of radiation, which suggests a biological interaction of AuNPs with cells could be the driver of radiosensitization. Additionally, AuNPs failed to radiosensitize two other cancer cell lines (DU145 and L132 cells) under identical conditions. The baseline radiosensitivity of all cells was similar; therefore, this suggests a cell-type-specific, biological mechanism of action rather than increased radiation dose deposition.

Innate immune responses may further enhance the anti-cancer efficacy of AuNP radiosensitization. Janic et al. investigated the use of mercaptosuccinic acid (MSA)-coated 4 and 14 nm AuNPs as potential therapeutic agents for breast cancer [12]. Human MDA-MB-231 cells were implanted into the flanks of nude mice, and tumors were allowed to grow to an average volume of 433 mm^3^. Tumors were directly injected with 100 µg of each size of AuNPs. Tumors were irradiated with a 15 Gy dose at 160 kVp source 24 h after AuNP injection. Both 4 nm and 14 nm AuNPs significantly decreased tumor growth compared to untreated controls, and the effect was similar to radiotherapy alone. When AuNPs were combined with radiation, tumor growth was further decreased, and 14 nm AuNPs outperformed 4 nm AuNPs for radiotherapy enhancement as indicated by improvements in tumor growth delay and overall survival. The examination of tumor tissue post-treatment showed increased levels of calreticulin, a key indicator of immune-stimulating damage-associated molecular patterns. Consistent with this, radiotherapy combined with AuNP treatment significantly increased macrophage infiltration. The authors concluded that the underlying mechanism involved not only AuNP-induced cytotoxicity but also immunological changes, which needs to be explored in more depth [12].

Despite extensive preclinical research, AuNPs have not yet been tested in humans as radiosensitizers. One limiting factor is that different sizes, shapes, and surface coatings all significantly impact radiosensitization, often with conflicting results, and it remains unclear how best to optimize these variables [38]. AuNP-mediated radiosensitization also varies depending upon radiation dose, X-ray energy, timing between nanoparticle delivery and radiation delivery, and in different cell lines in vitro as well as in different animal models. The lack of optimal materials, radiation exposure parameters, and an understanding of which model systems are most predictive of outcomes continues to hamper the clinical translation of AuNPs.

### 4.2. Hafnium Oxide Nanoparticles

In contrast to AuNPs, HfONPs have had greater success in translation from preclinical studies and into clinical trials. This in large part is due to focused research on a single type of HfONP developed by Nanobiotix. These particles, called NBTXR3, consist of a 50 nm core of HfO_2_ functionalized by a negatively charged phosphate coating. Like gold, the selection of hafnium as a material for use in radiation sensitization was driven by its high atomic number, which enables a high-probability interaction with ionizing radiation in the keV range, and the nanoparticles themselves are chemically inert in physiological solutions. In addition, the specific physicochemical features of NBTXR3 were selected to enable a high volume of distribution and retention in tumors following injection, to drive cancer cell uptake by endocytosis, and to minimize toxicity [50,54]. They are designed specifically for intratumoral injection and are not suited for intravenous use. Following injection into the tumor, NBTXR3 remains within the tumor throughout a multi-week radiotherapy course (5–7 weeks) so that one injection is enough to achieve dose enhancement for multiple rounds of radiation [32,58]. Once treatment is concluded, the nanoparticles can be removed through surgical resection of the tumor mass, though any minute fractions that escape into the systemic circulation likely are sequestered by the liver and spleen rather than being cleared through renal filtration [59]. Initial preclinical studies with NBTXR3 conducted by Maggiorella and colleagues in HT1080 cells (fibrosarcoma) estimated that dose enhancement ratios at 4 Gy were 1.4 for LINAC irradiation (6 MeV) and 1.8 for cobalt-60 irradiation (1.3 MeV) in vitro [54]. Although the dose of NBTXR3 needed to achieve these effects was not reported, this result indicated radiosensitization effects were greater when using lower energy X-rays, where photoelectric effects occur with higher probability. In the same study, they subsequently performed several experiments in vivo. First, the distribution of NBTXR3 was examined after injection into HT1080 tumor xenografts using X-ray micro-computed tomography (microCT). The images showed that nanoparticles distributed throughout the tumor volume and persisted with little change for the entirety of the 14-day study. In the second set of experiments, mice bearing HT1080 tumors were injected intratumorally with NBXTR3 (64 g/L solution; injection volume was not reported), and 24 h after injection the tumors were exposed to 4 or 8 Gy of radiation using a cobalt-60 source (1.3 MeV). Immediately after irradiation, the mice were euthanized, and tumors were dissociated into single cells then plated for clonogenic growth. Based on these data, an in vivo radiation dose enhancement ratio of 1.5 was calculated. The particles themselves did not affect the clonogenic growth of HT1080 cells. To further validate radiosensitization, mice bearing A673 Ewing’s sarcoma tumor xenografts were intratumorally injected with NBXTR3 as above and then exposed to 15 Gy of radiation using a cobalt-60 source (1.3 MeV). Kaplan–Meier curves showed a significant increase in the median survival time for NBTXR3 activated by 15 Gy exposure in comparison with 15 Gy alone (31 versus 25 days respectively). Mice bearing HCT116 colorectal carcer xenografts were injected with NBTXR3 as above and then exposed to 8 Gy of radiation as a single 8 Gy fraction or two 4 Gy fractions using an iridium-192 source (0.38 MeV). Under these conditions, tumor regression and long-term survival (up to 120 days) were observed for over two thirds of the mice that received NBTXR3 and radiation for both treatment schedules. The mice in all other groups (untreated, radiation alone, and NBTXR3 alone) did not survive beyond 70 days, indicating that NBTXR3 combined with radiation greatly improved treatment outcomes in mice. Although direct comparisons of the radiosensitizing effects of NBTXR3 cannot be made between the three tumor treatment models used in the Maggiorella study [54] due to variations among radiation sources and doses, the greatest benefit was observed for tumors irradiated with the iridium-192 source, which emits X-rays with an average energy of 0.38 MeV. It is likely that a portion of these X-rays were in the low keV range where photoelectric effects contribute to increased radiation dose deposition.

As with AuNPs, immune responses may further enhance HfONP-mediated radiosensitization [10]. Immune checkpoint inhibitors (ICIs) that block Programmed Death 1/Programmed Death Ligand 1 (PD1/PDL1) interactions or Cytotoxic T-Lymphocyte-Associated Protein 4 (CTLA4) activity have proven effective for the treatment of some cancers, but many fail to respond due to inherent or acquired resistance [60]. To address this issue, NBTXR3 with high- and low-dose radiation in combination with ICIs was tested in a murine, anti-PD1-resistant 3445QR lung cancer model with the goal of generating long-term anti-tumor immune memory [10]. Larger primary and smaller secondary tumors were implanted into the hindlimbs of mice. The primary tumor was injected with NBTXR3 (60.8 mg/mL in a volume equivalent to 25% of the tumor volume). The primary tumor was exposed to 36 Gy delivered in 12 Gy fractions daily for the three days immediately following the NBTXR3 injection. Radiation was delivered by an X-RAD 225Cx small-animal irradiator which produces a peak output of 225 keV X-rays. Some secondary tumors did not receive NBTXR3, but a subset of secondary tumors were irradiated with two 1 Gy fractions (2 Gy total) in parallel to the treatment of the primary tumors. Mice also received anti-PDL1 and anti-CTLA4 ICIs. The combination of NBTXR3 with 36 Gy directed to the primary tumor plus ICIs improved tumor control and reduced the number of spontaneous lung metastases, but ultimately all mice died within 65 days. However, long-term tumor control was achieved in mice receiving the combination of NBTXR3 with 36 Gy to the primary tumor plus ICIs plus 2 Gy directed at the secondary tumor. When the surviving mice were re-challenged by implanting 3445QR cells, the tumors failed to grow. These results suggest that the use of NBTXR3 nanoparticles in combination with radiotherapy and ICIs enhances anti-tumor immune response and provides long-term immune memory, which can be beneficial in the treatment of anti-PD1-resistant lung cancer.

Clinical studies in humans show that NBTXR3 enhances radiotherapy outcomes for soft tissue sarcoma [54] and head and neck cancer [33], and there are ongoing trials for additional types of cancer. Despite these impressive results, it is still unclear what drives the efficacy of HfONPs as radiosensitizers. Radiation dose enhancement estimates based on a Monte Carlo simulation under irradiation with 6 MeV X-rays show a dose deposition enhancement factor of 1.14 at a 10% mass ratio of NBTXR3 in water (100 mg/mL) [54]. For clinical applications, NBTXR3 is delivered by direct infusion into a tumor at a fixed concentration of 53.3 mg/mL in a volume equal to 10% of baseline tumor volume. Assuming homogeneous distribution in the tumor following infusion, this would result in a mass to volume ratio of 5.3 mg/mL (roughly a 0.5% mass ratio assuming 1 mL of tissue weighs 1 g). Therefore, based on estimates from simulations, the clinical dose of NBTXR3 would be expected to have little effect on radiation dose deposition, and thus little to no effect on treatment outcomes. However, even in high energy beams, a fraction of the X-rays are low energy, and simulations may underestimate the contribution of the interaction of these lower energy X-rays with the nanomaterials. Furthermore, the uptake of HfONPs by endocytosis leads to extremely high local concentrations of HfONPs in endosomes and other vesicles; therefore, localized dose enhancement in regions near these vesicles may be far greater than would be predicted from models assuming homogeneous distributions of nanoparticles across the entirety of the targeted volume. Another possibility is that there are specific features of the target cells that render them sensitive to even small increases in dose deposition. This possibility is supported in part by studies examining the radiosensitizing effects of NBTXR3 on a panel of nine human cancer cell lines [50]. It was observed that NBTXR3 was most effective at radiosensitizing cancer cell lines with greater inherent sensitivity to radiation alone. More research is needed to determine the relative contributions of each of these possible drivers of HfONP radiosensitization.

### 4.3. Silver Nanoparticles

Unlike AuNPs and HfONPs, AgNPs are known to be cytotoxic on their own. However, not all cells are equally sensitive to AgNP-induced cytotoxicity, and some cancers may be specifically vulnerable to AgNP cytotoxicity [61]. Recent studies have investigated the differential radiosensitizing effects of silver nanoparticles on cancer cells compared to non-cancer cells. In one study, clonogenic survival assays demonstrated that AgNPs significantly enhanced the sensitivity of breast cancer cells to ionizing radiation, while normal breast epithelial cells displayed relatively lower sensitivity to this combination therapy [51]. There were also differences in the relative sensitivity of different subtypes of breast cancer to AgNP-mediated radiosensitization. Doses of AgNPs as low as 1 μg/mL significantly increased the effectiveness of ionizing radiation (delivered using an orthovoltage source at 180 kVp) by roughly two-fold at a 2 Gy dose in triple-negative breast cancer (TNBC). For luminal A breast cancer cells (MCF-7) a higher dose of 5 μg/mL of AgNPs was necessary to produce a similar magnitude of radiosensitization to that observed in the TNBC cells. It required an even greater dose of AgNPs (10 μg/mL) to radiosensitize normal breast epithelial cells (MCF-10A), indicating that AgNPs may be particularly effective for radiosensitizing TNBC without harming normal breast tissue [51]. Similar selective radiosensitizing results were observed using other types of AgNPs, including triangular AgNPs roughly 90 nm in diameter [56].

AgNPs are also effective for radiosensitization using MeV X-ray irradiation. In one study, Liu et al. investigated the capacity of AgNPs to radiosensitize intracranial C6 gliomas in rats [62]. In this study, low doses (10 μg and 20 μg) of AgNPs were administered via stereotactic intratumoral injection followed by a single 10 Gy dose of ionizing radiation at 6 MeV energy 24 h later. Rats treated with 10 μg or 20 μg of AgNPs in combination with radiation exhibited significant extensions in median survival times, 100.5 and 98 days respectively, representing over a 5-fold increase compared to control groups. Furthermore, long-term survival (200-day cure rate) was achieved in 41.7% and 38.5% of animals in the 10 μg and 20 μg groups in combination with radiation, respectively. In contrast, neither radiation alone nor AgNPs alone provided significant survival benefits, with mean survival times ranging from 16.1 to 24.5 days and no observed cures. Importantly, no adverse effects were noted following nanoparticle administration, suggesting a favorable safety profile [62].

In direct comparison, AgNPs outperformed AuNPs as radiosensitizers for glioma in vitro and in vivo [53,55]. Specifically, U251 human glioma cells were exposed to AuNPs (10 μg/mL) or two doses of AgNPs (10 μg/mL and 5.48 μg/mL, the latter of which is equivalent to the molar concentration of AuNPs at 10 μg/mL) and irradiated with 6 MeV X-rays. At these nanoparticle concentrations, the dose enhancement ratio based on clonogenic survival of U251 cells was 1.23 for AuNPs. AgNPs outperformed AuNPs at both equal mass and equal molar concentrations (dose enhancement ratios 1.64 and 1.44 respectively). In agreement with previous studies [53], neither AuNPs nor AgNPs radiosensitized normal breast epithelial cells, indicating that these effects may be cell-type-specific.

AgNPs also outperformed AuNPs for radiosensitization in vivo following the intratumoral injection of AuNPs (10 μg) or AgNPs (10 μg or 5.48 μg) into mice bearing intracranial gliomas [53]. Approximately 24 h after the injection of nanomaterials, tumors were irradiated by 6 MeV X-rays with a dose of 8 Gy per mouse. Radiation alone increased mean survival time to 35.1 days compared to 24.1 days for the untreated mice. Mice receiving AuNPs or AgNPs alone had similar survival times to untreated mice. All mice treated with AgNPs or AuNPs combined with radiation had significantly longer survival times than control groups (61.7, 51.3, and 43.1 days for 10 μg AgNPs, 5.48 μg, and 10 μg AuNPs respectively). In addition, other studies showed AgNPs can radiosensitize tumors following intravenous injections [55]. One such study determined the ability of 18 nm, spherical AgNPs coated with polyvinylpyrrolidone (PVP), polyethylene glycol (PEG), or PEG terminated with a cancer-targeting aptamer (As1411) to target and radiosensitize C6 gliomas intracranially implanted in the brains of mice [55]. Mice were intravenously injected with each type of AgNP at a dose of 10 mg/kg. Six hours after injection, mice were irradiated by 6 MeV X-rays (6 Gy per mouse). The median survival times for mice treated with saline, PVP-coated AgNPs, PEG-coated AgNPs, or As1411-targeted AgNPs were 18, 19, 20 and 22 days respectively, and there was no significant difference among these four groups. Mice treated with saline, PVP-coated AgNPs, PEG-coated AgNPs, or As1411-targeted AgNPs plus irradiation therapy exhibited a significant increase in median survival times to 24, 30, 35 and 45 days, respectively. It is notable that radiosensitizing effects were observed at AgNP doses that were 200-fold less than those needed for radiosensitization by intravenously delivered AuNPs (10 mg/kg AgNPs versus more than 2 g/kg AuNPs [52]). The superior outcome of AgNPs as radiosensitizers compared to AuNPs and the cancer-specific radiosensitizing effects described above could not be predicted based upon the presumed physical mechanisms of dose enhancement; therefore, these studies support the existence of other underlying mechanisms that contribute to AgNP-mediated radiosensitization effects.

## 5. Biological Mechanisms of Nanoparticle Toxicity as Drivers of Radiosensitization

Because the observed DER often is greater than what would be predicted by alterations in DEF for irradiated cells containing nanomaterials, the underlying mechanisms by which high Z nanoparticles act as radiosensitizers are likely to involve biological mechanisms in addition to physical dose enhancement. For most radiation sensitization studies, the dose of the nanoparticles is minimized to reduce cytotoxicity, but it is still possible that sublethal effects occur in the absence of radiation. These effects may vary depending on the nanomaterial, the dose, the route of administration, the target cell type, and the model system. By increasing baseline levels of stress, the presence of nanomaterials within cells may reduce the dose of radiation needed to push cells over the lethal damage threshold. By understanding the specific mechanisms by which nanomaterials induce damage to cells, it will be possible to exploit this knowledge to match specific materials with the cancers that are most vulnerable to a particular type of stress. Of note, nanomaterials themselves can exhibit drug-like properties [63]. For example, anti-cancer mechanisms ascribed to AuNPs include the inhibition of oncogenic drivers including KRAS activation [64], insulin growth factor binding protein 2 (IGFBP2) signaling, and tumor-microenvironment crosstalk [65]. An emerging concept is harnessing the intrinsic cytotoxic activity of nanomaterials to modulate the dynamics of epithelial to mesenchymal transition (EMT) in cancer [66]. EMT is a conserved process involved in pathophysiological conditions including embryonic development, wound healing, and cancer. The EMT program represses epithelial characteristics such as apical/basal polarity and the expression of tight junction proteins (e.g., E-cadherin, claudins, and occludins). EMT is driven by transcription factors (e.g., ZEB1/2, TWIST1/2, SNAIL, and SLUG) and characterized by markers including N-cadherin, vimentin, and extracellular matrix proteins (e.g., fibronectin and collagen). Changes associated with EMT contribute to increased invasive capacity, heterogeneity within tumors, metastatic spread, and resistance to chemotherapy, immunotherapy, and radiation therapy [67,68,69]. Intriguingly, some nanomaterials may specifically target the mesenchymal cancer state. Exposure to AuNPs reduced the expression of mesenchymal markers in ovarian cancer in vitro, leading to the inhibition of tumor growth in vivo [70]. AgNPs selectively eliminated TNBCs [51,56,71,72,73], ovarian cancer [74], and non-small-cell lung cancer (NCSLC) [75,76] with a mesenchymal phenotype. AgNPs blocked the radiotherapy-induced expression of EMT markers in NSCLC [77]. Exposure to AuNPS reduced the expression of mesenchymal markers in ovarian cancer in vitro, leading to the inhibition of tumor growth in vivo [70]. Despite these observations, there is a large knowledge gap in the overall understanding of the drivers of nanomaterial cytotoxicity.

**Figure 2:**
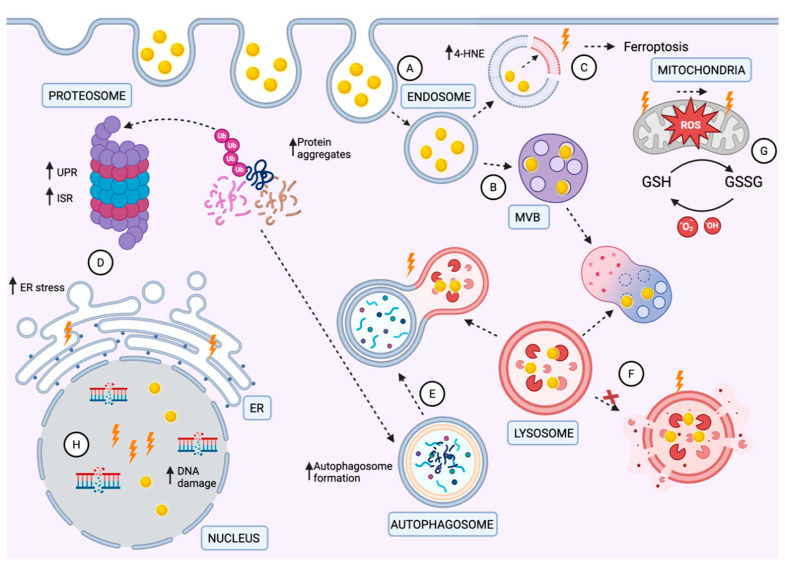
**Biological mechanisms of high Z nanoparticle radiation sensitization.** (**A**) Nanoparticles are taken up primarily by endocytosis. (**B**) Nanoparticles have been observed in early endosomes, late endosomes/multi-vesicular bodies (MVBs), lysosomes, and nucleus. (**C**) Nanoparticles induce lipid peroxidation and can lead to ferroptosis. (**D**) Nanoparticles induce proteotoxic stress leading to increased protein aggregation, unfolded protein response (UPR), integrated stress response (ISR) induction, and endoplasmic reticulum (ER) stress. (**E**) Nanoparticles block or induce autophagy depending on cancer context, including phenotype, duration of treatment, and dose. (**F**) Nanoparticles block lysosomal degradation. (**G**) Nanoparticles increase ROS generation by increasing hydrogen peroxide (H_2_O_2_) and the ratio of oxidized (GSSG) to reduced (GSH) glutathione. (**H**) Nanoparticles induce increased DNA damage.

Mechanistic studies are now unraveling some possible causes for the distinct responses of various cancers to different nanomaterials (Figure 2). Many studies conclude that increased ROS production is a driver of nanoparticle radiosensitization [17], but ROS plays a complex role in cancer. Global increases in ROS levels frequently do not correlate with the sensitivity of a specific cancer to ROS-inducing agents [78]. However, there are numerous variables to consider that are not captured by the assessment of steady-state ROS levels. These include the type of ROS produced, subcellular localization (e.g., mitochondrial vs. vesicular vs. cytosolic ROS), and the temporal dynamics of ROS production, reactivity, and degradation. Notably, the specific vulnerabilities of cancers to the types and localization of damage induced by ROS rather than the level of ROS itself may provide critical insight into which cancers may be most vulnerable to ROS-inducing nanoparticle radiosensitizers. Here, we examine two other forms of cellular damage potentially induced by ROS, proteotoxicity and lipid peroxidation, both of which are also induced by radiation [79], and discuss their potential roles in radiosensitization by high Z nanoparticles.

### 5.1. Proteotoxicity

Proteotoxic stress is emerging as an exploitable vulnerability for some cancers [80]. Proteotoxicity is a condition caused by the accumulation of misfolded and aggregated proteins in the cell, leading to protein homeostasis imbalance and subsequent cell death [81]. The acquired functional properties that enable cancer cells to survive often lead directly to proteotoxic stress [82]. Direct damage to proteins due to radiation and radiation-induced ROS contribute to the cytotoxic effects of ionizing radiation [83,84], and treatments that increase accumulation of damaged and misfolded proteins could enhance the effects of radiation. The induction of proteotoxic stress is just beginning to be explored as a potential toxicity mechanism for high Z nanoparticles [85], and this may be one of the mechanisms by which these types of nanomaterials exert their radiosensitizing effects. Critically, not all cancer cells are equally sensitive to proteotoxic stress inducers [80,86], and this heterogeneity may be why high Z nanomaterials are effective at radiosensitizing some cancers but not others.

Cells use the ubiquitin–proteasome system (UPS), lysosomes, and autophagy to reduce the burden of misfolded proteins [87,88,89]. The UPS is an ATP-dependent, non-lysosomal protein degradation mechanism in cells whereby proteins are tagged with ubiquitin and delivered to proteasomes for degradation [90]. Lysosomes degrade various intracellular and extracellular macromolecules so they can be recycled and reused [91,92]. Autophagy is a critical process in which damaged cellular components are sequestered in double-membrane-bound compartments and degraded after fusion with a lysosome [93]. This allows for the removal of damaged organelles, protein aggregates, and intracellular pathogens that would otherwise be cytotoxic [94]. Lysosomes participate in tumorigenesis by helping cancer cells respond to environmental stress [91]. During cancer development and progression, the demand for nutrients and energy needed for cancer cells increases greatly [91,92], and thus some cancers may be vulnerable to treatments that inhibit lysosome function. Although there are no reports of nanomaterials specifically affecting the function of proteosomes, nanomaterials can damage lysosomal membranes, impacting the degradation of misfolded proteins. AgNPs have been shown to induce autophagy [95,96,97] or block autophagic flux and induce lysosome dysfunction [11,98,99]. Depending on cell type, dose and formulation, and duration of treatment with AgNPs, either pro-survival autophagy [95,100] or pro-death autophagy was induced [11,98,99]. Mishra et al. investigated the impact of 10 nm, 50 nm, and 100 nm AgNPs on the autophagosome–lysosomal pathway and inflammasome activation in HepG2 cells, a human liver cancer cell line commonly used for hepatotoxicity studies [84]. The study revealed that smaller AgNPs caused a significant disruption of autophagic flux, possibly due to lysosomal membrane destabilization and functional impairment. Skalska et al. investigated the effects of orally ingested AgNPs on the adult rat brain [101]. The results showed an accumulation of autophagosomes, suggesting impaired autophagic flux due to lysosomal inefficiency. Morphological analysis revealed structural damage to lysosomes, further confirming their dysfunction. The disruption of lysosomal integrity and autophagic flux has also been shown following AuNP treatment [102]. A study by Li et al. demonstrated that AuNPs are internalized by the MRC-5-lung-fibroblast-caused formation of autophagosomes, as observed through transmission electron microscopy and evidenced by the upregulation of autophagy-related proteins, including microtubule-associated protein 1 light chain 3 (MAP-LC3) and autophagy gene 7 (ATG7) [103]. Less is known about the effects of HfONPs on autophagosome and lysosome function. However, the entrapment of a high concentration of HfONPs in endo/lysosomal compartments following uptake has been reported to increase lysosomal damage following radiation exposure, releasing lysosomal contents into the cytosol and potentially increasing proteotoxicity by decreasing protein degradation [104].

There are multiple other mechanisms cells use to mitigate proteotoxic stress. The integrated stress response (ISR) reduces protein synthesis while boosting protein degradation capabilities to alleviate proteotoxic stress [72,105]. Four distinct stress-sensing kinases can initiate the ISR: double-stranded RNA-dependent protein kinase (PKR), PKR-like ER kinase (PERK), heme-regulated eIF2α kinase (HRI), and general control non-de-repressible 2 (GCN2). Although they are each activated by different types of stress, all initiate the ISR through phosphorylation of the alpha subunit of eukaryotic translation initiation factor 2 (eIF2α) on serine 51. This inhibits the translation of most mRNAs and simultaneously activates an alternative transcriptional program that increases protein folding and degradative capacity. However, if the burden of misfolded proteins exceeds a cell’s capacity to recover, the ISR can drive cell death because the prolonged phosphorylation of eIF2α increases the expression of C/EBP Homologous Protein (CHOP), which induces apoptosis. The activation of the ISR leading to increased CHOP expression was observed in AgNP-treated TNBC cells, but not in luminal A or normal breast epithelial cells [73,106]. Based on these observations, it is possible that differences in tolerance to proteotoxicity may account for some of the observed differences in the radiosensitizing effects of AgNPs in TNBC cells and the lack of sensitization in luminal A breast cancer and normal breast cells [51]. Multiple studies also link AgNP exposure to the activation of endoplasmic reticulum (ER) stress as indicated by the unfolded protein response (UPR). Briefly summarized, the UPR is initiated by three ER-bound stress-sensing proteins, PERK, which can activate the ISR, activating transcription factor 6α/β (ATF6) and inositol-requiring enzyme 1α/β (IRE1) [107]. Some studies show the activation of all three arms of the UPR [108,109] following AgNP exposure, but other studies [51,71,72,76] do not demonstrate the activation of all arms of the UPR. Proteomic analysis of lung cancer cells with differing sensitivity to AgNPs indicated that in AgNP-sensitive lung cancer cells, but not in AgNP-insensitive ones, there was decreased activity of eIF2 and eIF4 and a substantial decrease in overall protein synthesis, consistent with ISR activation [76]. Noël et al. explored the sublethal effects of AuNPs on human neutrophils [110]. They observed the activation of all three arms of the UPR, which suggests that AuNPs can disrupt ER homeostasis and cause proteotoxic stress.

Although proteotoxic stress increases following exposure to some nanomaterials, responses are heterogeneous. Variables include differences in cell types, nanoparticle dosing, and time points. Some reports show that cells with high baseline stress on the proteostasis system exhibit increased sensitivity to small molecules that induce ER stress [80,111]. It is plausible that this feature could drive sensitivity to nanomaterials. Differences in nanoparticle uptake may be a contributing factor, but previous studies show that the sensitivity of a specific cell line to AgNPs is not proportional to the mass of AgNPs taken up by the cells, indicating that other factors may be more important [112]. Furthermore, it was found that AgNPs induced the ISR only in mesenchymal, claudin-low TNBC, but not in epithelial, basal-like TNBC or normal breast epithelial cells [71]. Mesenchymal subtypes of breast cancer cells are reported to produce excessive amounts of extracellular matrix (ECM) proteins, such as fibronectin and collagen, which sensitizes them to treatments that induce proteotoxicity due to a high baseline on proteostasis systems [72]. The treatment of breast cancer cells with a small molecule PERK inhibitor or ER chaperone mimetic (4-phenylbutyrate) reduced AgNP-induced apoptosis in breast cancer cells [105], which provides additional support for proteotoxicity as a key mechanism of action for AgNPs.

Overall, there is substantial evidence that proteotoxic stress increases following high Z nanoparticle exposure. The precise biological features that render some cells more sensitive than others to proteotoxicity induced by various nanomaterials, and what role this plays in radiosensitization, remain to be determined.

### 5.2. Lipid Peroxidation and Ferroptosis

Lipid peroxides are generated when ROS or free radicals react with polyunsaturated fatty acids (PUFAs) in cell membranes. Lipid peroxidation destabilizes cell membranes, disrupts signaling pathways, and damages other cellular components. The accumulation of lipid peroxides is a hallmark of a form of programmed cell death called ferroptosis, which involves the iron-catalyzed generation of free radicals that cause the catastrophic oxidation of phospholipids. Many types of inorganic nanoparticles, including those made from silver [72,113], iron [114,115], titanium dioxide [116], silica [117], cobalt NPs [118], or manganese [119], induce lipid peroxidation in a variety of cell types.

Emerging evidence suggests that lipid peroxidation plays a role in mediating the cytotoxic effects of AgNPs by disrupting membrane integrity and triggering oxidative stress-related cell death pathways [120]. In their 2020 study, Paciorek et al. investigated the extent and implications of lipid peroxidation in cells exposed to AgNPs, revealing that the peroxidation of PUFAs in cellular membranes is a primary mechanism of AgNP-induced toxicity [113]. They demonstrated that AgNP exposures for 24 h lead to dose-dependent increases in lipid peroxidation, as measured by the accumulation of well-established lipid peroxide degradation products including 4-hydroxynonenal (4-HNE) and malondialdehyde (MDA). These highly reactive aldehydes form protein and DNA adducts, impairing the function of critical biomolecules and signaling cascades. In particular, 4-HNE was shown to covalently modify thiol-containing proteins and enzymes involved in redox homeostasis, thereby amplifying ROS accumulation and disrupting antioxidant responses [113]. Some AuNP-treated cells also exhibited significant increases in lipid hydroperoxides [103]. Western blot analyses confirmed the presence of MDA protein adducts, and there was an upregulation of antioxidant enzymes and other oxidative stress response genes, suggesting an attempt to counteract the induced stress. Furthermore, levels of lipid peroxidation in cancer cells treated with HfONPs (NBTXR3) combined with radiation were reported to be significantly higher than those achieved with radiation alone [104].

Few studies have examined lipid peroxidation and ferroptosis as mechanisms by which high Z nanoparticles enhance radiosensitization, and it is not yet known if this is a general mechanism or if it is cell-type- or material-dependent. Additionally, not all cells are equally sensitive to lipid peroxidation and ferroptosis. Cells with membranes enriched with PUFAs, especially those containing arachidonic acid or adrenic acid, are more susceptible to ferroptosis, and the environmental availability of lipids further influences ferroptosis sensitivity [121]. The expression of EMT markers also correlates with the sensitivity of cancer cells to agents that induce lipid peroxidation [122,123]. Notably, studies investigating the cytotoxic effects of AgNPs on a panel of TNBC cell lines with different molecular subtypes [124] revealed that mesenchymal-like, claudin-low breast cancers (CLBCs) were more sensitive to AgNPs than epithelial-like basal-like breast cancer (BLBC) at doses that did not affect normal breast epithelial cells [106]. Mechanistically, the heightened sensitivity of CLBC cells to AgNPs was attributed to increased lipid peroxidation, which led to the accumulation of reactive lipid species, such as 4-hydroxynonenal (4-HNE). It is unclear whether this led to ferroptosis [72], but these results support the idea that high Z nanomaterials could be acting as radiosensitizers by a mechanism that includes increasing lipid peroxidation.

## 6. Remaining Challenges of Nanoparticle Use in Radiation Treatment

**Figure 3:**
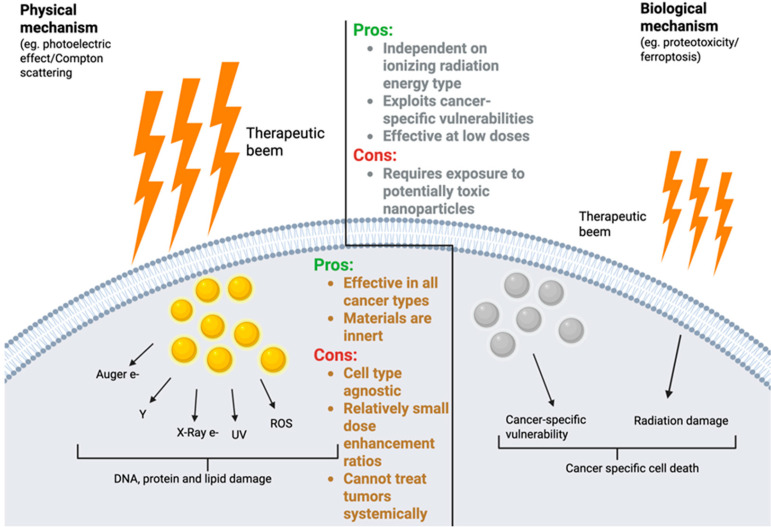
Summary of the complementary contributions of physical mechanisms and emerging biological mechanisms by which high Z nanoparticles act as radiosensitizers. While physical mechanisms emphasize the production of secondary electrons and reactive species leading to generalized DNA damage, biological mechanisms exploit cancer-specific vulnerabilities to enhance cell death in a more targeted manner. This distinction highlights the potential clinical advantages of leveraging biological pathways, such as effectiveness at low doses and independence from radiation type, enabling more effective and tumor-selective combination therapies, though challenges like nanoparticle toxicity remain.

Radiation plays a critical role in cancer treatment, but even the most advanced modalities have shortcomings due to the need to balance high-dose, cancer-killing efficacy with minimal toxicity to healthy tissue. Issues include the shifting of tumors due to respiration or digestive tract motion resulting in decreased on-target and increased off-target exposures. Furthermore, both inherent and acquired resistance, which are dependent upon the genetic makeup of the cancer, further limits efficacy. The drive to improve outcomes specifically in situations where dose escalation is not possible and current dosing is ineffective motivates the development of high Z nanomaterials as radiosensitizers. The physical mechanisms of dose enhancement based upon photoelectric effects or Compton scattering equally affect both normal and cancer cells, and therefore the challenge remains to achieve specificity in radiation delivery and dose enhancement. Thus, despite the potential of high Z nanoparticles in enhancing local dose deposition, achieving wide-spread clinical utility faces significant hurdles, primarily centered on delivery kinetics and long-term biocompatibility [125]. As outlined in Figure 3, understanding the biological drivers of radiation sensitization may improve outcomes by enabling the selection of the optimal type of high Z nanomaterials for the treatment of a specific subset of cancers. For example, as discussed in Section 5.2, the use of high Z nanomaterials to increase lipid peroxidation may be extremely effective and selective for the radiosensitization of mesenchymal-like TNBCs known to be vulnerable to ferroptosis. However, better approaches are needed to deliver nanoparticles to tumors in hard-to-reach sites, to target disseminated/metastatic tumors, and to identify biomarkers to define which patients are most likely to respond to specific nanomaterials.

Achieving therapeutic concentrations of high Z nanoparticles within tumors remains difficult. This is partly due to the heterogeneity of the EPR effect and elevated interstitial fluid pressure, which restrict nanoparticle penetration to the tumor periphery [126]; therefore, direct injection is the preferred means of nanoparticle delivery for radiosensitization. For example, HfONPs are limited to direct intratumoral administration because they are prone to aggregation in physiological solutions, and to achieve radiosensitization, a substantial mass of nanoparticles must be delivered to the tumor [127]. The standard formulation of NBTXR3 consists of a 53.3 mg/mL suspension, and doses as high as 25% of basal tumor volume are needed to achieve a therapeutic effect in mice [128] and 42% in humans [129]. In a clinical setting, the treatment of a 10 cm^3^ tumor would translate to 2.5 mL of a 53.3 mg/mL (133.25 mg) suspension of the current clinical formulation of NBTXR3, a dose which could not feasibly be reached by intravenous infusion, in which more than 1% of the administered dose rarely reaches the tumor [14]. AuNPs are considered suitable for systemic delivery, but most investigations still struggle to demonstrate high level tumor accumulation, with various studies estimating only 0.7–5% of the injection reaching the tumor [130]. Although the kinetics depend on the specific type and dose of AuNPs, they typically exhibit a biphasic blood clearance profile after intravenous injection with an initial first phase half-life of a few hours and an extended second phase with a half-life of up to several days [131]. As with most other types of nanoparticles, AuNPs inevitably are sequestered by the reticuloendothelial system, particularly the liver and spleen [130], which raises concerns regarding off-target toxicity and systemic clearance. However, within the therapeutic dosing range, there are no significant toxicity observations after AuNP treatment in animals [132,133], and AuNPs have been tested in humans with generally good safety profiles for multiple types of material and administration routes [134]. AgNPs appear to be effective as radiosensitizers at the lowest doses among the three nanomaterials discussed in this review. However, there is more debate regarding the potential toxicity of AgNPs, which in part is due to the frequent failure of investigators to separate highly toxic, dissolved silver cations (Ag+) from AgNPs [71]. As with other nanomaterials, the systemic administration of AgNPs raises concerns regarding long-term bioaccumulation in the liver, spleen, and kidneys, which can lead to chronic inflammation or organ damage [135]. Despite these concerns, studies by us and others using AgNPs free from Ag+ for the treatment of cancer [73] or viral diseases [136] show that AgNPs at 4–6 mg/kg delivered systemically for multiple weeks induce therapeutic responses including tumor regression and long-term survival without overt toxicity. In agreement with this, a direct comparison of the toxicity of citrate-coated AuNPs (10 mg/kg intravenous (IV) weekly for 8 weeks) and citrate-coated AgNPs (5 mg/kg IV weekly for 8 weeks), which roughly corresponds to a 1:1 ratio of silver and gold atoms, indicated that neither AuNPs nor AgNPs were toxic at these doses, though tissue discoloration and enlarged spleens were apparent [137]. These studies indicate that there is a safe dosing window for the intravenous delivery of high Z nanomaterials.

All three types of nanomaterials discussed here have progressed into clinical trials. Although current versions of HfONPs are not suitable for intravenous injection, AuNPs and AgNPs are actively being investigated for systemic and intratumoral injection for use as antivirals or in photothermal therapy. As summarized in Table 2, each of these nanomaterials has the potential to induce or enhance specific types of toxicity which may contribute to their efficacy as radiosensitizers.

There remain many variables to consider when designing and selecting nanoparticles as radiation sensitizers. In addition to the core materials, the size, shape, and surface functionalization are all key parameters that affect tumor accumulation, cell uptake pathways, and cytotoxicity. It is difficult to draw conclusions regarding the role of nanoparticle physicochemical features in cytotoxicity and radiosensitization processes without detailed examination of the delivery kinetics to cells and tumors, uptake mechanisms, and subcellular localization. This will require the development of standardized in vitro and in vivo models to better study and compare the role of the physicochemical features of different nanomaterials in driving specific biological and physical mechanisms of radiosensitization across research groups.

Abscopal effects of radiation have been described anecdotally in patients, but the true potential of radiation to enhance immunotherapy has not been fully exploited. We highlighted several studies in which high Z nanomaterials appeared to increase immune responses to treated cancers following radiation exposure [12,60]. Immunotherapy now represents a key arm for cancer therapy, and future studies of high Z nanomaterials need to examine interactions with immunotherapy, particularly for cancers that currently do not respond to treatments.

As more knowledge is gathered by researchers on the biological mechanisms driving the tumor-specific toxicity of high Z nanoparticles that contribute to radiosensitization, the focus can begin to shift to identifying biomarkers that distinguish cancers with heightened sensitivity to a particular type of nanomaterial from those that lack sensitivity. By enabling clinicians to choose the right material for the right cancer, biomarker-driven treatment selection will both enhance treatment efficacy and minimize adverse effects. However, to fully understand these underlying mechanisms, there is a critical need to transition toward developing standardized in vitro and in vivo platforms for establishing the reproducible benchmarks necessary to move nanoparticle-aided radiotherapy toward clinical implementation.

## 7. Conclusions

This review highlights the emerging understanding that the radiosensitizing mechanism of high Z nanoparticles is derived from more than just physical dose enhancement. Biologically driven mechanisms of toxicity play a role in radiosensitization. These mechanisms, such as proteotoxic stress, autophagy disruption, and lipid peroxidation, may allow for greater selectivity toward malignant tissue while minimizing collateral damage to surrounding healthy cells. The use of nanomaterials to selectively induce proteotoxic stress or ferroptosis as a therapeutic strategy for cancer treatment and the role these mechanisms play in nanomaterial-based radiosensitization are largely unexplored. The synergy between nanoparticle-mediated cytotoxic effects and ionizing radiation also has the potential to reduce both nanoparticle dose and radiation exposure, thereby enhancing therapeutic indices. Currently, to achieve a sufficient dose of high Z nanoparticles for radiosensitization, direct injection of nanoparticles into tumors is necessary to achieve sufficiently high doses for the physical mechanisms of radiosensitization to be effective. By understanding cancer-specific vulnerabilities and correctly matching these to nanomaterials that exploit each vulnerability, it may be possible to substantially decrease the doses needed for radiosensitization, opening the possibility for the systemic delivery of nanoparticles to enable the treatment of anatomically inaccessible and later-stage tumors. Importantly, the biological mechanisms of radiosensitization are not dependent upon the type of radiation administered, but rather the type of damage that radiation causes. Therefore, these materials would have application in combination with proton beam or neutron therapy. Lastly, recent evidence underscores the potential of metal nanoparticles as potent radiosensitizers that simultaneously prime the host immune system. The ability of this combined modality to elicit an immune response suggests a synergistic potential between nanoparticle-enhanced radiotherapy and immunotherapy. As we understand more about the mechanisms of high-Z-nanoparticle-mediated radiosensitization, we may be able to develop biological profiles identifying patients who are likely to benefit most from a specific nanomaterial, paving the way toward precision nanomedicine in radiation oncology.

## Figures and Tables

**Table 1:** Experimentally observed radiation therapy enhancing effects of high Z nanoparticles.

Reference	Nanoparticle Type/Dose	Cancer Model	X-Ray Source/Dose/Energy	Effect of Nanomaterials on Radiotherapy
[52]	AuNP; 1.9 nm spheres; 1.35 or 2.7 g/kg injected intravenously	EMT-6 syngeneic breast tumors in mice	Orthovoltage X-ray generator/30 Gy/250 kVp	Long-term survival (>1 year post-treatment): 86% or 50% survival for mice treated with 2.7 or 1.35 g/kg of nanoparticles plus radiation versus 20% survival for radiation alone and 0% for no treatment
[49]	AuNPs (Auravist); 1.9 nm spheres; 12 mM (approx. 2.36 mg/mL)	MDAMB231 cells in vitro	LINAC/4 Gy/160 kVp, 6 MeV, or 15 MeV	Dose enhancement ratio: 1.41, 1.29, 1.16 for 160 kVp, 6 MeV, and 15 MeV X-rays respectively based upon clonogenic growth
		DU145 cells in vitro	LINAC/4 Gy/160 kVp, 6 MeV, or 15 MeV	Dose enhancement ratio: 0.92, 1.13, 1.12 for 160 kVp, 6 MeV, and 15 MeV X-rays respectively based upon clonogenic growth
		L132 cells in vitro	LINAC/4 Gy/160 kVp, 6 MeV, or 15 MeV	Dose enhancement ratio: 1.05, 1.08, 0.97 for 160 kVp, 6 MeV, and 15 MeV X-rays respectively based upon clonogenic growth
[53]	AuNPs; 15 nm spheres; 10 µg/mL	U251 cells in vitro	LINAC/8 Gy/6 MeV	Dose enhancement ratio: Up to 1.23 based upon clonogenic growth
	AuNPs; 15 nm spheres; 10 µg injected directly into tumor	U251 brain tumor xenograft in mice	LINAC/8 Gy/6 MeV	Median survival time: 43.1 days for radiation plus nanoparticles versus 35.1 days for radiation alone based on Kaplan–Meier analysis
[12]	AuNPs; 4 nm spheres; 100 µg injected directly into tumors	MDA-MB-231 breast tumor xenografts in mice	Orthovoltage X-ray generator/15 Gy/160 kVp	Median survival time: 30 days for radiation plus nanoparticles versus 30 days for radiation alone based on Kaplan–Meier analysis
	AuNPs; 14 nm spheres; 100 µg injected directly into tumors	MDA-MB-231 breast tumor xenografts in mice	Orthovoltage X-ray generator/15 Gy/160 kVp	Median survival time: 26 days for radiation plus nanoparticles versus 21 days for radiation alone based on Kaplan–Meier analysis
[54]	HfONP (NBTXR3); 50 nm spheres; dose not reported	HT1080 cells in vitro	Cobalt-60/4 Gy/1.3 MeV (average); LINAC/4 Gy/6 MeV	Dose enhancement ratio: Up to 1.8 for Cobalt-60 and 1.4 for LINAC based upon clonogenic growth
	HfONP (NBTXR3); 50 nm spheres; 64 mg/mL injected directly into tumor; volume not stated	HT1080 tumor xenografts in mice	Cobalt-60/4 or 8 Gy/1.3 MeV (average)	Dose enhancement ratio: Up to 1.5 for ex vivo assessment of clonogenic growth of cells derived from irradiated tumors
	HfONP (NBTXR3); 50 nm spheres; 64 mg/mL injected directly into tumor; volume not stated	A673 tumor xenografts in mice	Cobalt-60/15 Gy/1.3 MeV (average)	Median survival time: 31 days for radiation plus nanoparticles versus 25 days for radiation alone based on Kaplan–Meier analysis
	HfONP (NBTXR3); 50 nm spheres; 64 mg/mL injected directly into tumor; volume not stated	HCT116 tumor xenografts in mice	Iridium-192/8 Gy/0.38 MeV (average)	Long-term survival (>120 days post-treatment): Over 66% survival for mice treated with nanoparticles plus radiation versus 0% survival for radiation alone
[10]	HfONP (NBTXR3) 50 nm spheres; 60.8 mg/mL injected directly into tumor in a volume equivalent to 25% of the tumor volume	344SQR syngeneic primary and secondary tumors in mice	Orthovoltage X-ray generator)/36 Gy primary tumor; 2 Gy secondary tumor/225 keV	Median survival time: Mice receiving the combination of radiation, immune checkpoint inhibitors, and nanoparticles survived 131 days versus 33.5 days for radiation and immune checkpoint inhibitors without nanoparticles
[51]	AgNPs; 20–30 nm spheres; 1–10 µg/mL	MDA-MB-231, BT549, MCF-7, and MCF-10A cells in vitro	Orthovoltage X-ray generator/0 to 4 Gy/300 keV	Dose enhancement ratio: Up to 1.5–2.0 for triple negative breast cancer cells (MDA-MB-231 and BT549) treated with 1 µg/mL of nanoparticles plus radiation; 2.0 for luminal A breast cancer (MCF-7) treated with 5 µg/mL of nanoparticles plus radiation; 2.0 for normal breast (MCF-10A) treated with 10 µg/mL of nanoparticles plus radiation
	AgNPs; 20–30 nm spheres; 0.2 μg/mm^3^ injected directly into the tumor	MDA-MB-231 breast tumor xenografts in mice	Orthovoltage X-ray generator/4 Gy/300 keV	Tumor growth inhibition: Significant decrease in tumor growth compared to untreated mice was observed for mice treated with nanoparticles plus radiation but not for nanoparticles or radiation alone
[53]	AgNPs; 15 nm spheres; 10 µg/mL	U251 cells in vitro	LINAC/8 Gy/6 MeV	Dose enhancement ratio: Up to 1.64 based upon clonogenic growth
	AgNPs; 15 nm spheres; 10 µg/mL	U251 brain tumor xenograft in mice	LINAC/8 Gy/6 MeV	Median survival time: 61.7 days for radiation plus nanoparticles versus 35.1 days for radiation alone based on Kaplan–Meier analysis
[55]	AgNPs; 18 nm spheres with HER-2 aptamer; 30 μg/mL	C6 cells in vitro	LINAC/0 to 8 Gy/6 MeV	Dose enhancement ratio: Up to 1.62 based upon clonogenic growth
	AgNPs; 18–20 nm spheres with HER-2 aptamer; 10 mg/kg injected intravenously	C6 brain tumor xenografts in mice	LINAC/6 Gy/6 MeV	Median survival time: 45 days for radiation plus nanoparticles versus 24 days for radiation alone based on Kaplan–Meier analysis
[56]	AgNPs; 90 nm triangular prisms; 1.25 or 2.5 µg/mL	MDA-MB-231 and MCF10 cells in vitro	Orthovoltage X-ray generator/0 to 4 Gy/300 keV	Dose enhancement ratio: Up to 1.6 or 2.6 for triple negative breast cancer cells (MDA-MB-231) treated with 1.25 or 2.5 µg/mL of nanoparticles plus radiation respectively but only 0.87 of 1.3 for MCF-10A cells under equivalent conditions based upon clonogenic growth

**Table 2:** Clinical status and toxicity mechanism of hafnium oxide, gold, and silver nanoparticles for radiation sensitization.

Nanoparticle	Intended Route of Administration	Clinical Stage	Key Toxicity Mechanisms
HfONP (NBTXR3)	Intratumoral injection (local)	CE Mark approval in Europe as a medical device for soft tissue sarcoma; not yet approved by US FDA but currently in multiple Phase III trials.	DNA damage; lysosomal damage; lipid peroxidation
AuNP	Intravenous injection (systemic) Intratumoral injection (local)	Not clinically approved; currently in Phase I clinical trials for radiotherapy combined with photothermal therapy.	Inhibition of oncogenic drivers; EMT inhibition; autophagy disruption; lipid peroxidation
AgNP	Intravenous injection (systemic) Intratumoral injection (local)	Clinically approved for topical applications for wound healing; Phase I clinical trials completed for intravenous use for treatment of SARS-CoV-2; not currently in clinical trials for radiation sensitization.	EMT inhibition; autophagy disruption; lysosomal damage; proteotoxic stress; lipid peroxidation; DNA damage

## Data Availability

Data sharing is not applicable. No new data were created or analyzed in this study.
